# Comparative Analysis of the Impact of Urolithins on the Composition of the Gut Microbiota in Normal-Diet Fed Rats

**DOI:** 10.3390/nu13113885

**Published:** 2021-10-29

**Authors:** Ali Khalaf Al Khalaf, Abdulrasheed O. Abdulrahman, Mohammed Kaleem, Suza Mohammad Nur, Amer H. Asseri, Hani Choudhry, Mohammad Imran Khan

**Affiliations:** 1 Department of Biochemistry, Faculty of Science, King Abdulaziz University, Jeddah 21589, Saudi Arabia; a_dietitian@hotmail.com (A.K.A.K.); mkaleem002@stu.kau.edu.sa (M.K.); snur0001@stu.kau.edu.sa (S.M.N.); ahasseri@kau.edu.sa (A.H.A.); hchoudhry@kau.edu.sa (H.C.); 2 Centre for Artificial Intelligence in Precision Medicines, King Abdulaziz University, Jeddah 21589, Saudi Arabia

**Keywords:** gut microbiota, polyphenols, urolithins, rats, metabolism, diseases, functional food

## Abstract

The gut microbiota consists of a community of microorganisms that inhabit the large intestine. These microbes play important roles in maintaining gut barrier integrity, inflammation, lipid and carbohydrate metabolism, immunity, and protection against pathogens. However, recent studies have shown that dysfunction in the gut microbiota composition can lead to the development of several diseases. Urolithin A has recently been approved as a functional food ingredient. In this study, we examined the potentials of urolithin A (Uro-A) and B (Uro-B) in improving metabolic functions and their impact on gut microbiota composition under a metabolically unchallenged state in normal rats. Male Wistar rats (*n* = 18) were randomly segregated into three groups, with Group 1 serving as the control group. Groups 2 and 3 were administered with 2.5 mg/kg Uro-A and Uro-B, respectively, for four weeks. Our results showed that both Uro-A and B improved liver and kidney functions without affecting body weight. Metagenomic analysis revealed that both Uro-A and B induced the growth of Akkermansia. However, Uro-A decreased species diversity and microbial richness and negatively impacted the composition of pathogenic microbes in normal rats. Taken together, this study showed the differential impacts of Uro-A and B on the gut microbiota composition in normal rats and would thus serve as a guide in the choice of these metabolites as a functional food ingredient or prebiotic.

## 1. Introduction

The human microbiome consists of a community of microorganisms, including bacteria, archaea, protozoa, and viruses, living in a symbiotic relationship with the host in various parts of the body [1]. However, most of the microbes live in the large intestine, with an estimated 10^11–12^ bacterial per gram of the content [2]. This community of microbes plays an essential physiological role relating to metabolism, immunomodulation, nutrient extraction, digestion, and vitamin synthesis [3,4]. Interestingly, more than 60% of the bacterial species in the gut are specific to each human [1], with diet, hygiene, host genotype, and geographical location influencing the gut microbiota composition [5]. Other influencing factors include prebiotics, probiotics, and polyphenol [6].

Polyphenols are natural compounds found in plants. They are present in abundant levels in foods such as fruits, teas, vegetables, cereals, and coffee. The dietary intake of polyphenols from time to time has been linked to beneficial health outcomes relating to oxidative stress modulation, cardiovascular function, inflammation, and lipid profile, among others [7]. Following their ingestion, polyphenols are considered as xenobiotics by the body with a relatively lower bioavailability than the micro and macronutrients. Besides, lower molecular weight polyphenols such as the procyanidins are easily absorbed in the small intestine without further conjugation. The higher molecular weight polyphenols, on the other hand, might become built up in the large intestine where they are acted upon and are bio-transformed by the gut microbiota [8,9]. Hence, the potential health benefits of polyphenol-containing food substances might depend on their conversion into a more bioavailable form by the gut microbiota [7]. Therefore, the potential positive effects of polyphenol-rich foods might be dependent on biotransformation into more bioavailable forms by intestinal bacteria, potentially modulating related health benefits.

Dietary polyphenols exist in a mutual relationship with the gut microbiota, where unabsorbed polyphenols are metabolized into a more bioavailable metabolite [10]. However, the mechanisms utilized by polyphenols to modulate the gut microbiota are still poorly understood. Nonetheless, they are thought to stimulate the growth of beneficial bacteria or inhibit the growth of harmful ones [11]. In view of this, current research efforts are geared towards manipulating the gut microbiota via dietary or therapeutic interventions with the sole aim of achieving a healthy microbiome [12]. Hence, determining the gut microbiota composition of disease-free individuals is important in understanding and establishing the differences in the composition between normal and diseased conditions [13].

Urolithins are secondary metabolites obtained from the gut microbial action on foods and fruits that are rich in ellagic acid (EA) or ellagitannin (ET) such as pomegranates, grapes, nuts, and berries [14]. Following their ingestion and under the influence of the gut microbiota, the ellagitannins undergo hydrolysis, decarboxylation, and dihydroxylation reactions to form a series of urolithin metabolites, including urolithin A, B, C, D, and Iso-Urolithin A [15,16]. These urolithin metabolites are more bioavailable than the parent polyphenol compounds. They have been detected in various target tissues and thus have been suggested as being responsible for the biological activities linked to the intake of ellagitannin-containing foods [17]. However, not all individuals are able to produce urolithins from the ingestion of ellagitannin rich-food sources. Due to this, urolithin-producing individuals have been placed into three groups or metabo-types. First, Uro-A-producing individuals are placed under metabo-type A. Second, Uro-A, IsoUro-A, and Uro-B-producing individuals are classified as metabo-type B. Third, urolithin non-producing individuals are placed under metabo-type 0 [16,18]. Previous studies have reported various biological activities for the urolithins. These include anti-cancer [19], antioxidant, anti-inflammatory, antiglycation [17] and modulation of lipid levels [20,21], among others.

The majority of recent studies involving the urolithins have focused on their effects on various metabolic disorders including diabetes [22,23], obesity [24,25], and cardiovascular disease [21,26]. Recently, chemically synthesized Uro-A has been shown to be safe in humans when taken orally [27] and has also been suggested for use as a functional food ingredient [28]. However, not all individuals possess the microbiome for producing urolithins. In this study, we examined the impact of administration of Uro-A and Uro-B on metabolically unchallenged rats fed on a normal diet.

## 2. Materials and Methods

### 2.1. Animals and Treatment

Male Wistar rats (*n* = 18) weighing 320–330 g were obtained from the animal house of the King Fahd Medical and Research Centre, King Abdulaziz University, Jeddah, Saudi Arabia, and were made to acclimatized to the lab environment for 7 days. During this period, animals were maintained on a normal diet, 22 ± 2 °C temperature and a 12/12 light off and on cycle. The study involving lab animals was conducted in accordance with the Declaration of Helsinki, and the protocol was approved by the Ethics Committees, Department of Biochemistry, King Abdulaziz University, Jeddah, Saudi Arabia (No.: 192060117). Following acclimatization, animals were randomly divided into three groups (*n* = 6) as follows:

Group 1 (ND) was maintained on a normal diet for four weeks.

Group 2 (NDA) was maintained on a normal diet and received intraperitoneal (IP) injection of Uro-A at a dose of 2.5 mg/kg [29] four times a week for four weeks.

Group 3 (NDB) was maintained on a normal diet and received an IP of Uro-B at a dose of 2.5 mg/kg [29] four times a week for four weeks.

**Figure 1:**
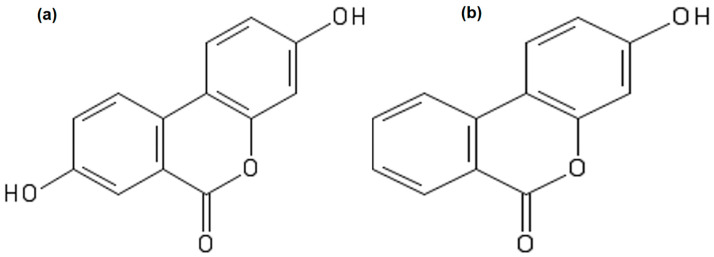
Chemical structure of urolithins. Urolithin A (**a**), urolithin B (**b**).

Urolithin A and B (Figure 1) were purchased from BLD Pharma, Shanghai, China, and 20 mg/mL stock solutions of each of these compounds were prepared by dissolving them in dimethyl sulfoxide (DMSO) and subsequently stored at 4 °C protected from light. Prior to IP injection, phosphate-buffered saline was used to dilute a small volume of the stock solution, and the required amount of urolithin was then injected into the animals. Untreated animals in the control group received the same volume of the vehicle.

At the end of the experiments, food was taken away from the animals overnight and they were subsequently euthanized under diethyl ether anesthesia. Blood was withdrawn from the retro-orbital plexus, and then the animals’ intestinal contents were retrieved under aseptic settings and stored at −80 °C until ready for analysis.

### 2.2. Determination of Serum Biochemical Parameters

Animals’ serum samples were obtained after centrifuging whole blood at 3000 rpm for 10 min. Thereafter, serum levels of urea and aspartate aminotransferase (AST) were assessed using a commercial kit (Crescent diagnostics, Jeddah, Saudi Arabia), according to the manufacturer’s instructions.

### 2.3. Sequencing of Bacterial 16S rDNA Gene and Data Processing

The total genomic DNA from the intestinal contents of the animals was extracted with the aid of a QIAamp Fast DNA Stool Mini Kit (Qiagen, Hilden, Germany) according to the manufacturer’s instructions and confirmed on a 1% agarose gel electrophoresis. The extracted DNA samples were subjected to 16S rDNA gene sequencing by targeting the V3 and V4 region with specific primers; 341F (5′-CCTACGGGNGGCWGCAG-3′) and 805R (5′-GACTACHVGGGTATCTAATCC-3′). After PCR amplification and purification, the purified amplicons were subjected to pyrosequencing on an Illumina MiSeq platform (Macrogen, Seoul, South Korea). Following the Illumina MiSeq data analysis, the sequences were assembled into an operational taxonomy unit (OTU) with a 97% similarity. The Chao1, Shannon, and ACE alpha diversity indices were analyzed using QIIME (version 1.9). The beta diversity was estimated with the use of the unweighted UniFrac distance metric, and the visualization of the principal coordinate analysis (PCoA) plot was performed using EMPEROR.

### 2.4. Statistical Analysis

The data obtained in this study have been presented as mean ± SE. Statistical analysis was achieved with a GraphPad Prism V6.0 software (GraphPad Software, San Diego, CA, USA) using either one-way ANOVA followed by Dunnett’s multiple comparisons test or Kruskal–Wallis test followed by Dunn’s multiple comparisons test. * *p* < 0.05 was taken as significant.

## 3. Results

### 3.1. Effects of Urolithin on the Final Body Weight, Liver, and Kidney Functions

**Figure 2:**
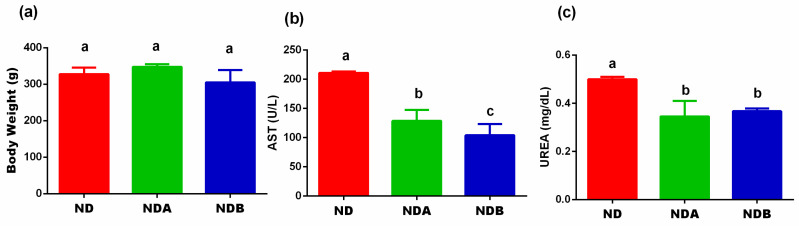
Effects of urolithins on body weight and organ function. Final body weight (**a**), AST. (**b**), Urea (**c**). ^a^^,b,c^ Data are expressed as mean ± SE (*n* = 6). Mean values bearing different letters are significantly different (*p* < 0.05).

First, we examined the effects of Uro-A and B on the final body weight of normal rats maintained on a normal diet and treated with the urolithin metabolites. As shown in Figure 2, there is no significant difference in the body weight between the normal diet-fed animals and the urolithin A treated rats fed on a normal diet. A similar result was obtained with the Uro-B treated rats as compared to animals maintained on a normal diet only. However, both Uro-A (*p* < 0.05) and Uro-B (*p* < 0.01) significantly reduced the serum level of AST. Furthermore, both Uro-A and Uro-B significantly (*p* < 0.05) attenuated the serum level of urea (Figure 2).

### 3.2. General Gut Microbiota Composition

**Figure 3:**
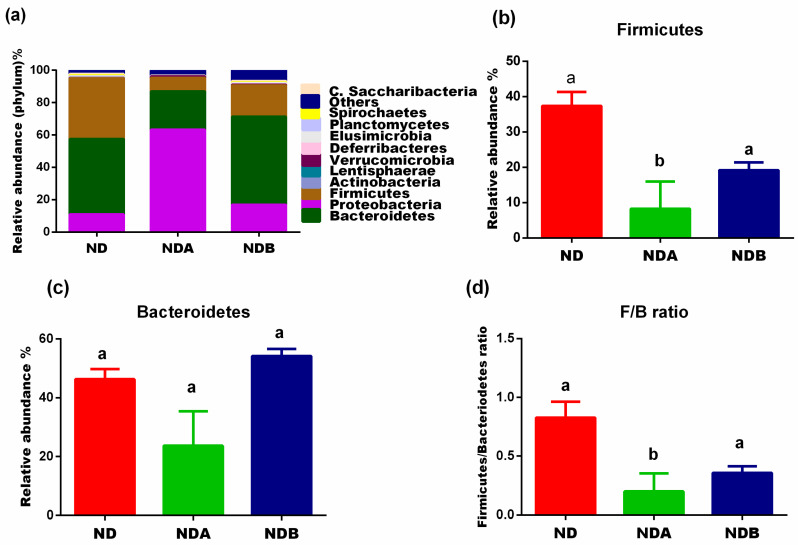
Effects of urolithins on the composition of the gut microbiota at the phylum level. Relative abundance at the phylum level (**a**), relative levels of the Firmicutes (**b**), relative levels of the Bacteriodetes (**c**), Firmicutes/Bacteriodetes ratio (**d**). ^a,b^ Data are expressed as mean ± SE. Mean values bearing different letters are significantly different (*p* < 0.05).

A total of 920,620 high-quality reads were obtained from the metagenomics amplicon of the extracted intestinal DNA, with the sequences grouped into 3998 operational taxonomic units. The Proteobacteria, Bacteroidetes, and Firmicutes made up 97.9% of the phyla, and the remaining 2.1% comprised of Actinobacteria, Lentisphaerae, Verrucomicrobia, Deferribacteres, Elusimicrobia, Planctomycetes, Spirochaetes, and Candidatus Saccharibacteria (Figure 3a). The Good’s Coverage revealed coverage of 99% (Table 1), which indicates that the majority of the samples’ bacterial content had been detected when compared to the literature database.

**Figure 4:**
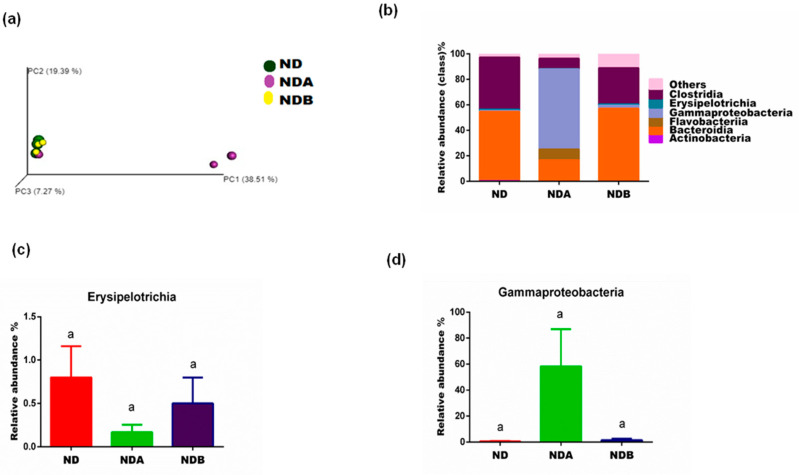
Effects of urolithins on the structure and composition of the gut microbiota. Principal coordinate analysis (**a**), relative abundance at the class level (**b**), relative levels of Erysipelotrichia (**c**), relative levels of Gamma-proteobacteria (**d**). ^a^ Data are expressed as mean ± SE. Mean values bearing different letters are significantly different (*p* < 0.05).

The beta diversity from the principal coordinate analysis (unweighted Unifrac) of the untreated animals in the control group and Uro-B treated group revealed that the gut microbes from these two groups clustered together. However, the gut microbiota from Uro-A treated animals showed that the bacterial clustered differently (Figure 4a). The alpha diversity index with Chao1 showed that there was no significant difference in the species diversity between the animals fed with a normal diet and Uro-B-treated animals. However, animals treated with Uro-A revealed a decrease in species diversity when compared to untreated animals fed on a normal diet. A similar observation was noted with the Shannon microbial diversity richness. Uro-A treated animals showed a decrease in microbial diversity when compared with animals fed on a normal diet. However, the microbial diversity was almost the same in animals administered with Uro-B when compared with animals fed on a normal diet (Table 1).

### 3.3. Gut Microbial Alteration in Animals

At the phylum level, compared with untreated animals, the population of Bacteroidetes decreased in Uro-A-treated rats. On the other hand, Uro-B administration to rats increases the population of the Bacteroidetes compared to the untreated control rats. Urolithin A and B administration to rats increased the levels of Verrucomicrobia and Proteobacteria as compared to the untreated control rats. In contrast, animals treated with both Uro-A and B revealed a decreased level of Firmicutes when compared with untreated control animals (Figure 3).

At the class level, the population of Erysipelotrichia and Clostridia decreased in animals treated with both Uro-A and Uro-B when compared with untreated control animals. A similar result was obtained with the population of Actinobacteria. The levels of these microbes were also reduced with the treatment with Uro-B when compared with untreated control rats. However, the level of Actinobacteria was completed lost with Uro-A administration (Figure 4).

**Figure 5:**
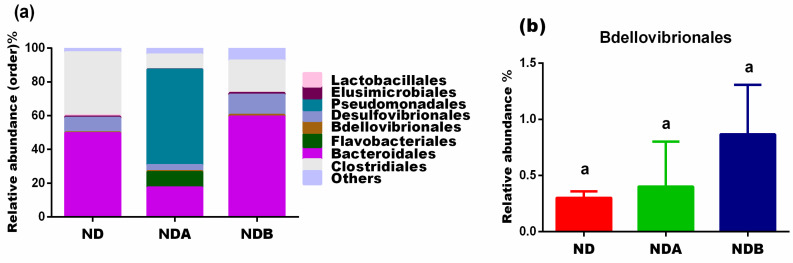
Effects of urolithins on gut microbial composition at the order level. Relative abundance at the order level (**a**), relative levels of the Bdellovibrionales (**b**). ^a^ Data are expressed as mean ± SE. Mean values bearing different letters are significantly different (*p* < 0.05).

At the order level, Uro-A administration to rats induced the growth of Flavobacteriales and Pseudomonadales as these bacteria were only observed in this group (Figure 5a). The levels of Lactobacillales and Clostridiales decreased in both Uro-A and Uro-B-treated rats when compared to the untreated control animals. However, Uro-A and Uro-B administration to rats increased the population of the Bdellovibrionales when compared to untreated control rats (Figure 5b). The levels of Elusimicrobiales were fairly the same in both Uro-B treated rats and the untreated control rats. However, Uro-A treatment decreased the levels of the Elusimicrobiales when compared to untreated animals in the control groups (Figure 5a).

**Figure 6:**
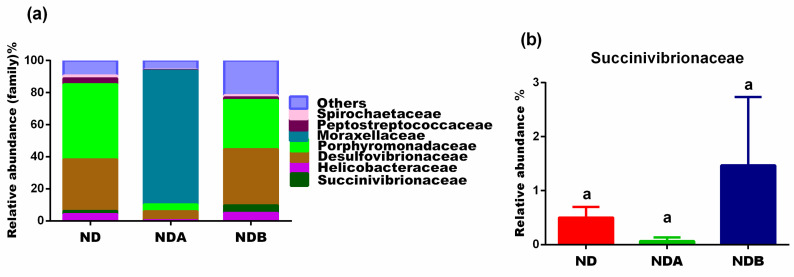
Effects of urolithins on gut microbial composition at the family level. Relative abundance at the family level (**a**), relative levels of Succinivibrionaceae (**b**). ^a^ Data are expressed as mean ± SE. Mean values bearing different letters are significantly different (*p* < 0.05).

At the family level, Uro-B administration to rats increased the levels of Succinivibrionaceae and Desulfovibrionaceae when compared with untreated animals maintained on a normal diet. Uro-A treatment, on the other hand, decreased the levels of these microbes (Figure 6). Compared with the untreated control group, both Uro-A and Uro-B treatment decreased the population of Peptostreptococcaceae. The levels of Spirochaetaceae were fairly the same in both Uro-B-treated animals and untreated animals in the control group. However, Uro-A decreased the levels of Spirochaetaceae when compared to the untreated animals in the control group (Figure 6).

**Figure 7:**
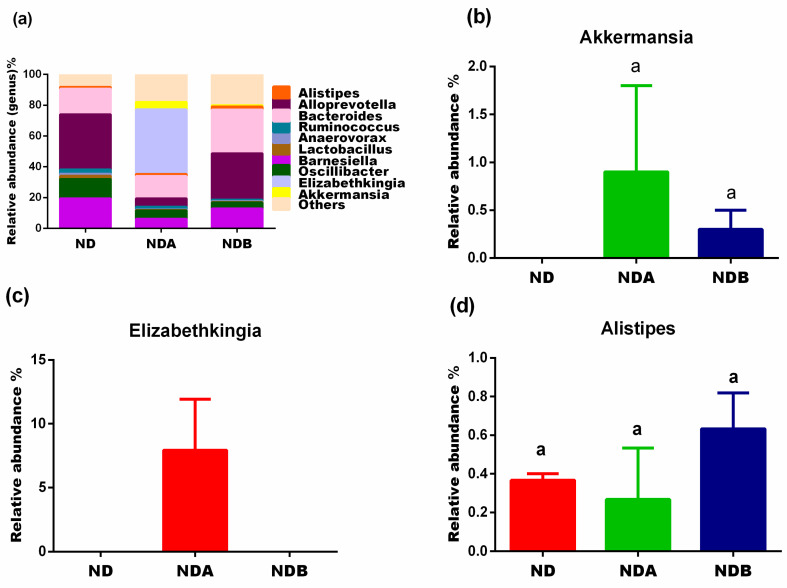
Effects of urolithins on gut microbial composition at the genus level. Relative abundance at the genus level (**a**), relative levels of Akkermansia (**b**), relative levels of Elizabethkingia (**c**), relative levels of Alistipes (**d**). ^a^ Data are expressed as mean ± SE. Mean values bearing different letters are significantly different (*p* < 0.05).

At the genus level, compared to untreated rats in the control group, both Uro-A and Uro-B treated rats showed a decreased levels of Barnesiella, Oscillibacter, Lactobacillus, Anaerovorax, and Ruminococcus. Furthermore, Uro-B treatment does not affect the levels of Alloprevotella, whereas Uro-A administration to rats decreased the levels of Alloprevotella when compared to untreated rats in the control group (Figure 7a). The levels of Bacteroides and Alistipes decreased following Uro-A treatment when compared to untreated animals in the control group. The levels of Bacteroides and Alistipes, however, were elevated following the administration of Uro-B. Interestingly, both Uro-A and Uro-B induced the growth of Akkermansia, the level of which was completely lost in the untreated animals in the control group (Figure 7).

## 4. Discussion

Dietary polyphenols such as anthocyanins, ellagitannins, and phenolic acids have received great attention for their potential role in mitigating many health challenges such as cardiovascular diseases and other age-related diseases [30]. However, due to their poor bioavailability, these natural compounds have been suggested to act mainly at the intestinal absorption level [31].

Before now, studies involving urolithins have mostly centered around disease models with the potential of using them as a new treatment or therapeutic regimen for human diseases. In this study, however, we compared the effects of two urolithin metabolites, Uro-A and Uro-B, and their effects on metabolically unchallenged normal rats. Our results showed that the administration of both Uro-A and Uro-B to normal rats has no effects on the bodyweight of rats. This result showed the specificity of urolithins on body weight and differentiated its effects on normal and obese rats. For instance, several previous studies have reported the bodyweight reduction potentials of the urolithins in obese rats [24,25]. Our results also showed that the treatment of animals with both Uro-A and B offered more protection to the liver and kidney (Figure 2).

The gut microbiota plays an essential role in the maintenance of the host’s metabolic status. An undisturbed gut microbiota facilitates the extraction of energy, modulation of the host’s immunity, and the generation of short-chain fatty acids [32]. Recent findings have considered the gut as having about a thousand microbes residing in the host’s gastrointestinal tract with just a few important bacterial species responsible for the metabolism of phenolic compounds [10]. The gut microbiota role in metabolizing dietary polyphenols could provide valuable insights into the health-promoting potentials of these compounds [33]. Several research efforts have been ongoing on the use of natural compounds to target the gut microbiota for the improvement of human health. This is achieved either through the use of prebiotics or probiotics to target the growth of beneficial bacteria or inhibit harmful ones [34].

The Firmicutes and the Bacteriodetes constitute the main bacteria at the phylum level, irrespective of the health status of the animal. It has been previously reported that the Firmicutes assist the host in food catabolism and the degradation of cellulose into fatty acid. They have also been shown to play an important role in the immune response through their action on the blockage of invading pathogenic bacteria and their protective effect against intestinal inflammation [35,36]. However, the Bacteriodetes, on the other hand, are butyrate producers and assist the host in maintaining a healthy gut. They play an essential role in bile acid metabolism and the biotransformation of harmful compounds. Studies have shown that the Bacteriodetes benefit the host by breaking down polysaccharides into glucose, thus providing nutrients for the host [37].

Indeed, the Firmicutes to Bacteroidetes (F/B) ratio has been previously shown to increase from birth to adulthood [38] and has been suggested as a critical index for the health status of the host [39]. A change in the Firmicutes/Bacteroidetes ratio is synonymous among different studies concentrating on diseases. For example, an increase in this ratio has been linked to aging [40] and obesity [41]. Another study also found an increase in the F/B ratio in hypertensive rats [42]. Thus, any intervention that lowers the F/B ratio could be an important strategy for the treatment of hypertension, aging, and other metabolic diseases. Interestingly, the F/B ratio was lower in the urolithin-treated animals when compared to the untreated animals in the control group (Figure 3d) and confirms previous studies on the beneficial effects of urolithins on aging [43,44] and cardiovascular functions [21].

Dietary polyphenols differ in their influence on the gut microbiota. They can either exert their antibacterial properties on certain bacteria, thereby limiting their growth or inducing other beneficial bacteria’s growth [45]. In this study, we also noted that the urolithins exerted their growth-inhibiting effects on certain bacteria while inducing the growth of others. For example, the Akkermensia genus (phylum Verrucomicrobia), which are oval-shaped, anaerobic, Gram-negative bacteria, are found in great abundance in the intestinal tract of humans and correlate inversely with different disease conditions [46]. They inhabit the intestinal mucosal layer, where they activate the metabolic and immune response in the host. They achieve this through the generation of short-chain fatty acid, leading to the simulation of goblet cells. The goblet cells produce mucus which is important in the preservation of the intestinal barrier integrity leading to a reduction in intestinal inflammation. The Akkermansia have also been shown to possess oxygen scavenging properties resulting in anerobic bacteria growth [45]. Accumulated evidence in the literature showed the association of an elevated abundance of A. muciniphila to its positive effects in different metabolic disorders, including diabetes, obesity, and metabolic syndrome [47]. The abundance of Akkermensia has also been noted in mice where they led to an increase in the expression of genes linked to immune response [48]. These studies suggest that an increased abundance of Akkermansia in the host intestine could offer a promising prospect in treating metabolic diseases. Interestingly, this genus is completely absent in the untreated animals fed on a normal diet and present only in the guts of the urolithins treated groups (Figure 7b). This study showed the potential of the urolithins to induce the growth of Akkermansia and further confirms a previous study showing that the induction of Akkermansia after the intake of pomegranate extract in humans [49].

Bdellovibrionales (phylum *Proteobacteria*) are found in the intestines of animals, including humans. They are gram-negative bacteria that function to control the population of other microbes. In order to survive, members of the Bdellovibrionales order prey on other gram-negative bacteria [49]. Previous studies reported using B. bacteriovorus as a biological therapeutic agent to target pathogenic bacteria [50,51]. However, low levels of B. bacteriovorus, a species in this order, have been found in the intestines of celiac patients and those with inflammatory bowel disease, resulting in the uncontrolled growth of bacteria [52,53]. This implies that a high abundance of Bdellovibrionales could be preventive against IBD and other inflammatory diseases since the microbes target the growth of pathogenic bacteria responsible for inflammation. Our studies showed that both Uro-A and Uro-B treatment increased the abundance of these microbes, and they could thus be used as prebiotic for targeting pathogenic bacteria. This study also confirms previous reports that showed urolithins’ antimicrobial potentials against pathogenic Yersinia enterocolitica [54].

Furthermore, the analysis of our results revealed that Uro-A administration to rats led to a decrease in the Chao1 and Shannon indices (Table 1), implying that the Uro-A decreased alpha diversity, which might be related to the antibacterial potentials of Uro-A. We observed that these antibacterial potentials of Uro-A are not specific to pathogenic bacteria. It also decreases the growth of other microbes of medical importance. Our results further revealed that Uro-B might be an excellent antibacterial prebiotic as compared to Uro-A. This is because while Uro-A increased the growth of some pathogenic microbes as compared to the untreated animals in the control group, Uro-B, on the other hand, decreased their growth. For example, the Elizabethkingia (phylum Bacteroidetes) is a genus of Gram-negative, non-spore-forming bacteria found all over the environment. These microbes are highly resistant to antibiotic treatment, and inappropriate use of antimicrobial treatment has been reported to be a risk factor for patients infected with Elizabethkingia [55]. The majority of the species in this genus are pathogenic to both animals and humans and have been shown to be responsible for the cause of fatal diseases, including meningitis, deafness, and brain abscess [56]. The 16s rDNA analysis of our results showed that this genus is completely absent in both the untreated animals fed on a normal diet and Uro-B treated animals. However, Uro-A treatment induced the growth of this bacteria (Figure 7c).

Furthermore, the Gammaproteobacteria class is made up of different groups of bacteria with varied phenotypic and metabolic properties. They are mainly chemoorganotrophs; however, some are chemolithotrophs or phototrophs. These microbes use the oxidation of iron, hydrogen, or sulfur to obtain their metabolic energy. Representative microbes from this class include Escherichia coli which has been reported to house many pathogens that affect humans, animals, and plants [57,58]. A growing body of evidence reports that inflammatory bowel disease arises due to the disturbances in the gut microbiome, including a reduction in gut bacterial diversity and an increase in the population of the Gammaproteobacteria [59]. The increased population of the Gammaproteobacteria in the gut has also been reported to the positively associated with metabolic syndrome, cancer, and inflammation [60] and may also contribute to chemoresistance with treatment involving gemcitabine [61]. Our results showed that Uro-B treatment prevented the growth of this bacteria, revealing its antibacterial potentials against diseases causing gut microbes such as Elizabethkingia and Gammaproteobacteria.

The Succinivibrionaceae family are Gram-negative, anaerobic and non-spore-forming bacteria. They are chemoorganotrophs, and members of this family possess the potentials to ferment glucose and other carbohydrates into succinate and acetate [62]. Previous studies reported that members of the Succinivibrionaceae are elevated in human immunodeficiency virus (HIV)-infected patients and have been proposed as a biomarker of immune recovery in these patients [63]. For example, there is an increased synthesis of some transport proteins such as the SLC5/6 proteins from this family during HIV infections. These SLC5/6 proteins are active transporters of molecules implicated in reducing inflammation and immune recovery in HIV-infected persons [63]. It can thus be inferred that an elevated abundance of the Succinivibrionaceae might play an essential role in immune recovery, especially in HIV-infected people. Our results showed that Uro-B treated rats increased the population of these microbes, but decreased with Uro-A administration when compared with untreated animals fed on a normal diet (Figure 6b). This result points to an important aspect of selecting the right prebiotics to target specific bacteria. The antibacterial potentials of Uro-A are not essential in this case since it led to the inhibition of the growth of important bacteria required for immune recovery and might thus be detrimental if used as a prebiotic by HIV-infected patients.

Similarly, Alistipes (phylum *Bacteroidetes*) are anaerobes mostly seen in the gut of healthy humans. Previous studies reported the dysbiosis of the Alistipes in several human and animal disease models, including cardiovascular diseases, liver disease, and colon cancer [64,65]. Liver diseases such as non-alcoholic fatty liver disease (NAFLD), non-alcoholic steatohepatitis, and cirrhosis have been linked to the microbiota-liver axis, suggesting that a change in the composition of the gut microbiota might be a likely cause of these diseases [66]. In a report examining the relationship between liver fibrosis and gut microbiota, the authors found a decreased composition of the Alistipes throughout the development of liver fibrosis [67]. A similar result was reported for liver cirrhosis in which a reduced composition of Alistipes was observed in affected patients in comparison with healthy individuals in the control group [68]. In this study, we observed an increased relative abundance of the Alistipes in Uro-B administered rats (Figure 7d). Like the Succinivibrionaceae, the abundance of the Alistipes reduced in Uro-A-treated rats fed on a normal diet. Compared to Uro-A, this result showed the potentials of Uro-B to increase the growth of Alistipes, which could be important in the prevention of liver cirrhosis and other liver diseases.

## 5. Conclusions

In this study, we examined the impact of administration of Uro-A and Uro-B on a metabolically unchallenged state in rats fed on a normal diet. We showed that both Uro-A and Uro-B did not affect weight gain in normal diet-fed rats. However, these metabolites enhanced liver and kidney functions. Furthermore, we showed that both Uro-A and Uro-B induced the growth of Akkermensia and increased the abundance of Bdellovibrionales, two important microbes which have positive impacts on different metabolic diseases and on the control of intestinal pathogens, respectively. Finally, we showed that Uro-A and Uro-B have varied impacts on Elizabethkingia, Gammaproteobacteria, Succinivibrionaceae, and Alistipes, whose dysbiosis have been implicated in different disease conditions. Taken together, this study showed the differential impacts of Uro-A and B on the gut microbiota composition in normal rats and would thus serve as a guide in the choice of these metabolites as a functional food ingredient or prebiotics.

## Figures and Tables

**Table 1:** Bacterial diversity from intestinal contents of animals.

Group	Good’s Coverage	Chao 1	Shannon
ND	99.61	635.9	7.16
NDA	99.86	268.6	3.08
NDB	99.65	592.0	6.71

## Data Availability

Data related to this study are contained within the article.
